# Sarcopenia, Obesity, Sarcopenic Obesity and Risk of Poor Nutritional Status in Polish Community-Dwelling Older People Aged 60 Years and Over

**DOI:** 10.3390/nu14142889

**Published:** 2022-07-14

**Authors:** Marika Murawiak, Roma Krzymińska-Siemaszko, Aleksandra Kaluźniak-Szymanowska, Marta Lewandowicz, Sławomir Tobis, Katarzyna Wieczorowska-Tobis, Ewa Deskur-Śmielecka

**Affiliations:** 1 Department of Palliative Medicine, Poznan University of Medical Sciences, 61-245 Poznan, Poland; marikamurawiak87@gmail.com (M.M.); ola.kaluzniak@gmail.com (A.K.-S.); mlewandowicz@ump.edu.pl (M.L.); kwt@tobis.pl (K.W.-T.); edeskur@ump.edu.pl (E.D.-Ś.); 2 Department of Occupational Therapy, Poznan University of Medical Sciences, 60-781 Poznan, Poland; stobis@ump.edu.pl

**Keywords:** sarcopenia, obesity, sarcopenic obesity, older adults, nutritional status, malnutrition

## Abstract

Poor nutritional status (PNS) is a modifiable factor determining abnormalities in body composition-sarcopenia, obesity, and sarcopenic obesity (SO). We aimed to assess the prevalence of these conditions and their association with PNS in 211 community-dwelling older adults. Sarcopenia was diagnosed based on the European Working Group on Sarcopenia in Older People 2 (EWGSOP2) recommendations. Obesity was diagnosed with the Percent Body Fat (>42% in women and >30% in men). Subjects fulfilling the criteria for obesity and concomitantly with reduced lower and/or upper limbs muscle strength and muscle mass (ALM/BMI < 0.512 in women and <0.789 in men) were classified as SO phenotype. Participants without obesity and sarcopenia were categorized as ‘normal’ phenotype. Nutritional status was estimated with the Mini Nutritional Assessment, and a score of <24 indicated PNS. In total, 49.8% participants had abnormal body composition (60.7% men and 42.5% women; *p* = 0.001). Sarcopenia, obesity, and SO were diagnosed in 10%, 32.7%, and 7.1% of subjects. PNS was found in 31.3% of the study sample. Its prevalence differed between phenotypes: 81% in sarcopenia, 60% in SO, 14.5% in obesity, and 28.3% in the ‘normal’ phenotype group (*p* = 0.000). Based on the results, abnormal body composition is prevalent in elderly subjects. Sarcopenia and SO are often associated with PNS.

## 1. Introduction

Physiological aging is associated with changes in body composition, such as progressive loss of muscle mass and redistribution of fat tissue [1,2]. These changes favor pathologies such as sarcopenia, obesity, and sarcopenic obesity (SO) and pose a threat to healthy aging [3,4,5]. The etiology of age-related changes in body composition is complex and poorly understood so far. A cascade of negative, mutually driven metabolic, hormonal, and myocellular mechanisms and lifestyle factors was suggested [5,6,7]. Age-related muscle mass loss results in a decrease in basal metabolic rate, which may increase fat mass. Fat tissue is an active endocrine organ, producing hormones and cytokines involved in systemic inflammation, oxidative stress, mitochondrial dysfunction, and insulin resistance [2,8,9]. An ectopic fatty infiltration of skeletal muscles, known as miosteatosis, reduces the effectiveness of muscular contraction and impairs muscle strength [10,11,12].

Poor nutritional status is an important lifestyle factor implicated in the etiology of body composition abnormalities. Impaired nutritional status in older adults may result from an inappropriate diet and socio-economic factors such as poverty or feelings of loneliness after the death of a spouse [13,14]. Older persons are at higher risk of impaired nutritional status [15]. In older individuals, malnutrition (quantitative and/or qualitative) often exacerbates age-related muscle mass loss, regardless of total body mass and Body Mass Index (BMI) [16]. Poor nutritional status impends successful aging. An inadequate intake of calories and protein may lead to sarcopenia [17,18]. Sarcopenia is associated with an increased health burden, including the risk of dependence, functional impairment, higher morbidity, and mortality [19,20]. In Europe, sarcopenia affects about 10% of community-dwelling older people and 29% of residents of long-term care facilities [21].

The aging of society coincides in many countries with a pandemic of obesity. Obesity and sarcopenia are both pathological body composition phenotypes and impaired nutritional status conditions. According to the Eurostat data from 2019, 52.7% of the EU citizens aged ≥65 years are overweight (BMI 25–29.9 kg/m^2^), including 16.5% of obese individuals (BMI ≥ 30 kg/m^2^) [22]. Obesity is a strong predictor of general health decline and quality of life deterioration in elderly subjects. Age-associated fat tissue redistribution favors visceral obesity and its complications, such as metabolic syndrome, type 2 diabetes, sleep apnea, and increased mortality [23,24].

The prevalence of sarcopenia and obesity increases with age. Thus, older subjects have a higher risk of coincidence of both conditions. Sarcopenic obesity is defined as a clinical–functional status combining sarcopenia and increased fatness. Some research data indicate that SO is not just a simple coincidence of the two pathological phenotypes but has a synergic effect of a cascade of unfavorable functional and metabolic factors [25,26,27]. Fatness and muscle atrophy enhance mutually, predisposing to comorbidity, immobilization, dependence, disability, low quality of life, and increased mortality [28,29,30]. It is estimated that SO affects 5–10% of the older population and is even higher in subjects over 80 years. The true prevalence of SO is challenging to assess, owing to the lack of widely accepted diagnostic criteria and low awareness of this relatively novel condition [31].

Due to their high prevalence in elderly subjects, demographic trends, and global population aging, pathological body composition phenotypes are an increasing challenge for health, social and health policies in many countries [32,33,34]. Our study aimed to assess the prevalence of sarcopenia, obesity, and SO in older Polish citizens living in the community and investigate the association between the Mini Nutritional Assessment questionnaire (MNA) score and these conditions. To the best of our knowledge, there are only a few papers assessing the risk of malnutrition based on a validated screening tool simultaneously in the three pathological body composition phenotypes [35,36,37,38,39]. Our study was the first research conducted in central Europe.

## 2. Materials and Methods

### 2.1. Study Design and Participants

We cross-sectionally analyzed data of 211 community-dwelling elderly subjects in the Greater Poland (Wielkopolska) voivodship in Poland. The data were gathered from October 2018 to November 2021. The study protocol was reviewed by the Bioethics Committee of Poznan University of Medical Sciences, Poland (Protocol No: 872/18). The inclusion criteria were: age ≥60 years, normal cognitive functions (Abbreviated Mental Test Score (AMTS) ≥ 7, lack of contraindications to body composition analysis with the bioimpedance method (BIA) (cardiac pacemaker, metal implants, peripheral oedemas), ability to maintain a standing position for height measurements and body composition analysis.

We used the AMTS [40] to exclude subjects with cognitive impairment. The test consists of 10 items scored 1 for each item. Patients with a total score ≥ 7 (out of 10 maximum), which indicates a lack of significant cognitive impairment, were included in the analysis.

### 2.2. Nutritional Status Assessment

The nutritional status was assessed with MNA questionnaire, as recommended by The European Society for Clinical Nutrition and Metabolism (ESPEN) [41]. All participants were screened with the 6-item MNA-Short Form (MNA-SF). Individuals with scores ≤11 (out of 14 maximum) were further investigated with additional 12 questions (MNA-Long Form; MNA-LF). The MNA-LF consists of 18 items covering four domains: anthropometrics (weight loss during the past three months, BMI, mid-arm circumference, and calf circumference); general health status (mobility, psychological stress/acute disease over the past three months, neuropsychological problems, place of living, number of prescription drugs, pressure sores or skin ulcers); dietary assessment (decline in food intake over the past three months, number of full meals eaten daily, consumption of protein, fruit and vegetables, fluid intake and mode of feeding) and self-view of nutritional and health status. The maximum MNA-LF score is 30 points. A score ≥24 points indicates normal nutritional status, 17–23.5 indicates being at risk of malnutrition, and <17 indicates malnutrition. The individuals who scored <24 points were considered to have PNS (malnourished or being at risk of malnutrition). All subjects were assessed with both MNA-SF and MNA-LF.

### 2.3. Body Composition Analysis

Body composition was analyzed using the bioimpedance method with an InBody 120 analyzer (Biospace Seoul, South Korea). Bioimpedance is an inexpensive and non-invasive method based on measurements of soft tissue resistance and reactance to low-intensity electric current (≤1 mA). The following parameters were assessed: body weight, BMI, fat tissue mass (FM), skeletal muscle mass (SMM), fat-free mass (FFM), percentage of body fat (PBF), total body water (TBW), and segmental fat-free mass (for five segments: upper limbs, lower limbs, trunk).

### 2.4. Sarcopenia Phenotype

Sarcopenia was diagnosed with the EWGSOP2 criteria, following the F–A–C–S algorithm (Find–Assess–Confirm–Severity Pathway) [42]. The SARC-F questionnaire was used as a screening tool. SARC-F consists of 5 questions assessing strength, assistance in walking, rising from a chair, climbing stairs, and falls. The maximum score is 10 points, and a score ≥4 suggests sarcopenia. The F–A–C–S algorithm in such cases recommends further diagnostics of sarcopenia (except for subjects with clinical suspicion of sarcopenia). In order to classify participants to particular body composition phenotypes, we completed diagnostics for sarcopenia in all subjects (regardless of the SARC-F score). To this end, we assessed upper and lower limb muscle strength, muscle mass, and physical capacity. Decreased muscle strength indicated probable sarcopenia. Low muscle strength combined with decreased muscle mass confirmed the diagnosis of sarcopenia. Physical capacity was used to assess the severity of sarcopenia.

Upper limb muscle strength was assessed with the handgrip strength test (HGS) using a dynamometer (Saehan, Changwon, Korea). The test was performed in a sitting position, with arms bent at 90 degrees. Two measurements were taken for each hand. The best result was compared with cut-off values recommended by the EWGSOP2: 16 kg for women and 27 kg for men. Results below these values indicated low upper limb strength [42].

Lower limb muscle strength was assessed with the Five-Repetition Sit-to-Stand test (5STS). We measured the time taken by the patient to complete five rises from a chair with arms crossed at the chest. Less than 15 s indicated reduced lower limb muscle strength [42].

Muscle mass was assessed with the BIA parameters. Following the EWGSOP2 criteria, we used the Appendicular Lean Mass (ALM) index, defined as the sum of the lean tissue in the arms and legs (kg) scaled to height squared (m^2^) [42]. We used the following cut-off values for low muscle mass: <5.6 kg/m^2^ in women and <7.4 kg/m^2^ in men [43].

Physical capacity was assessed with the 4 m usual walking speed test, performed on an unimpeded walkway, with marked turning points indicating the 4-meter distance. We measured the time necessary to cover this distance and calculated the walking speed. The result of <0.8 m/s suggested low physical capacity [42].

### 2.5. Obesity Phenotype

Obesity was diagnosed based on PBF assessed with the BIA method. The cut-off points for excessive adiposis were >42% in women and >30% in men [35,44,45].

### 2.6. Sarcopenic Obesity Phenotype

Sarcopenic obesity was diagnosed based on modified recommendations from the ESPEN and EASO (the European Association for the Study of Obesity group experts) [46]. The modification consisted of the replacement of the ALM/Weight for the muscle mass assessment with the ALM/BMI, commonly used in the diagnostic of SO in previous research. SO was diagnosed in participants concomitantly fulfilling the following criteria:

- Reduced muscle strength in lower and/or upper limbs to assess the upper limb muscle strength subjects performed HGS twice with each hand; the cut-off points were <16 kg in women and <27 kg in men; the lower limb strength was assessed with 5STS, with a cut-off point of >15 s [42];
- Increased fat mass (the cut-off points for excessive adiposis were >42% in women and >30% in men [35,44,45]);
- Reduced muscle mass based on the ALM/BMI index and cut-off points of <0.512 in women and <0.789 in men [47].

Based on the body composition phenotype, participants were classified into four subgroups: sarcopenia phenotype (sarcopenia without obesity), obesity phenotype (obesity without sarcopenia), SO phenotype (concomitance of sarcopenia and obesity), and normal phenotype (no obesity, no sarcopenia).

### 2.7. Covariates

The following data were taken for each participant: marital status (unmarried/married), residential pattern (living alone/living with a mate), education level (lower than primary/higher than primary), number of chronic diseases and number of regularly taken prescribed drugs.

### 2.8. Statistical Analysis

Statistical analysis was performed by sex and age (two age groups: younger old (60–74 years) and older old (≥75 years)). Categorical variables were presented as numbers (n) and percentages (%). Quantitative data were described with means and standard deviations. If appropriate, categorical variables were analyzed with Pearson′s Chi-squared test with Yates correction. The difference between the two age groups (younger old/older old) was assessed with the Student′s *t*-test for data with normal distribution and homogeneity of variance, the Cochran–Cox test for data with normal distribution, which failed the test for homogeneity of variance or Mann–Whitney U test for variables lacking normality. The differences between the four phenotype groups were assessed with analysis of variance (ANOVA)—in case the data fulfilled the assumption of normality and homogeneity of variance. The differences between pairs of groups were evaluated with Tukey posthoc test or Kruskal–Wallis test for data without normal distribution. *p*-value < 0.05 was considered significant. All statistical analyses were performed with STATISTICA 13 PL (Tibco Software, Poland).

## 3. Results

### 3.1. Characteristics of the Study Population

**Figure 1:**
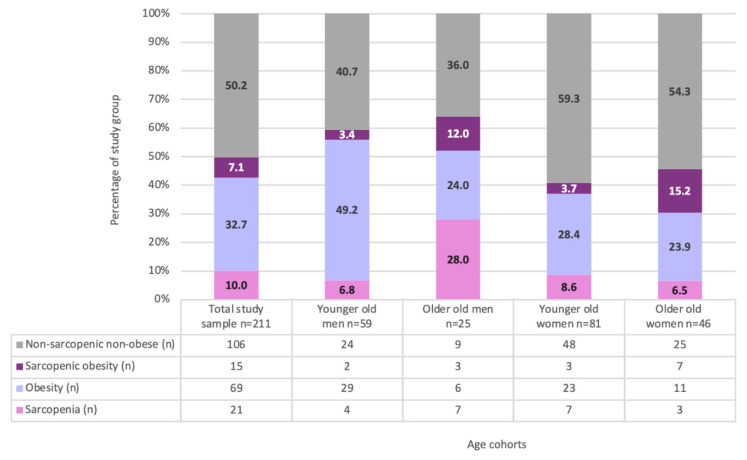
The prevalence of body composition phenotypes in total study sample and subgroups by sex and age cohorts.

We included data from 211 subjects aged ≥60 years (mean age 72.4 ± 7.0; 60.2% women). One out of three participants was living alone. Almost half of the study population (46.1%) was unmarried; the percentage of unmarried subjects increased with age, but the statistical significance was found in men only (19% in the age group 60–74 years vs. 40% in the age group 75 + years; *p* = 0.04). Over ninety percent of the study sample (93.2%) declared education level higher than primary; no significant differences between sex and age groups were observed. Data are presented in Table 1. Almost half of the study sample (49.8%) had abnormal body composition; it was more frequent in men than in women (60.7% vs. 42.5%; *p* = 0.001). Sarcopenia, obesity and SO was diagnosed in 10% (7.9% women and 13.1% men), 32.7% (26.7% women and 41.7% men), and 7.1% participants (7.9% women and 6% men), respectively. No differences between sex groups were found, except for obesity, which was more frequent in men (*p* = 0.02). The percentage of sarcopenia and SO was higher in the older age group (75+). Men 75+ had sarcopenia more frequently than younger ones (28% vs. 6.8%; *p* = 0.02), while women ≥75 years had SO four times more frequently than their younger counterparts (15.2% vs. 3.7%; *p* = 0.048). Obesity was more prevalent in younger age groups than in older ones, with a significant difference in men (49.2% in the age group 60–74 years vs. 24.0% in men 75+; *p* = 0.03). Detailed data are shown in Figure 1.

Based on the MNA-LF questionnaire, nearly one-third (31.3%) of the study sample had poor nutritional status. Malnutrition was rarely diagnosed (3.3%), and 85.7% of all malnourished subjects were women aged 60–74 years. Nutritional status tended to be worse in the older age group (not significantly). No significant differences between sex and age groups were observed for MNA domains. Detailed data are presented in Table 1.

### 3.2. Body Composition Phenotypes

One-way ANOVA revealed significant differences (*p* < 0.05) between four analyzed phenotypes for nearly all assessed parameters (Table 2a–c), except for AMTS (*p* = 0.07), and three socio-demographic parameters (sex, marital status and residential status) and III domain in the MNA-LF, assessing dietary habits (ns).

#### 3.2.1. Sarcopenia Phenotype

**Figure 2:**
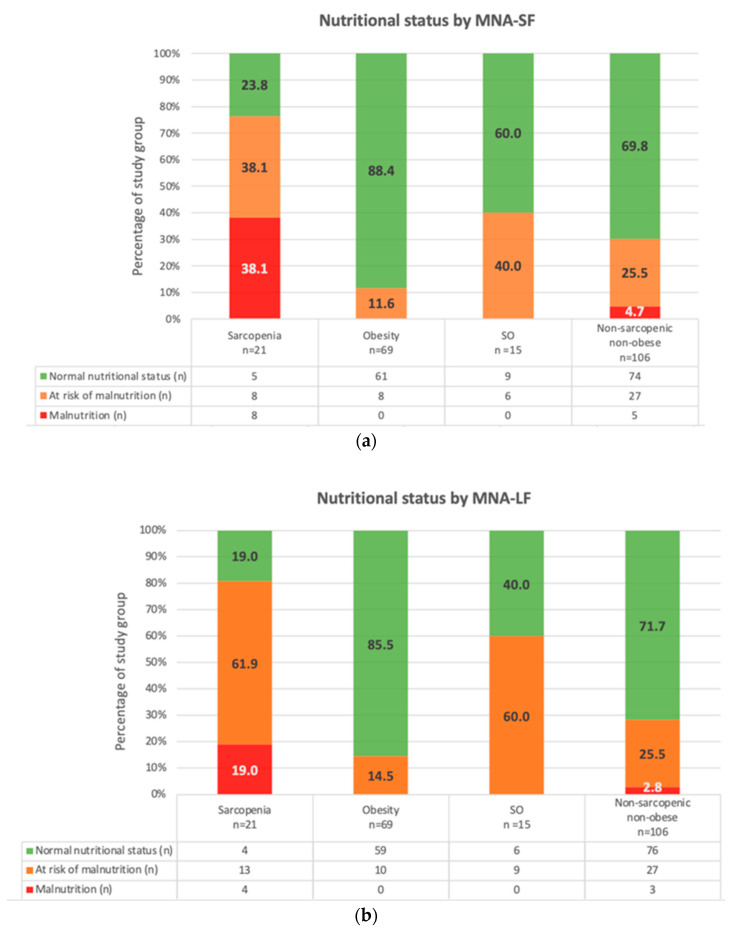
(**a**) Nutritional status assessed with MNA-SF by phenotypes groups. (**b**) Nutritional status assessed with MNA-LF by phenotypes groups. Note: *p* = 0.000 for comparisons between four analyzed phenotypes (one-way ANOVA).

Subjects with sarcopenia had the lowest body weight, BMI, and ALM of all phenotypes (Table 2b). Compared to subjects with a ‘normal’ phenotype, they had lower muscle strength in the upper limb (*p* = 0.0003), lower limb (*p* = 0.005), and decreased physical capacity (*p* = 0.04) (Table 2c). Most persons with sarcopenia had PNS. The percentage of subjects with PNS was higher in the sarcopenic phenotype group than in ‘normal’ and obese ones. Malnutrition was diagnosed in subjects with sarcopenia much more frequently than in the ‘normal’ phenotype group (eight times more frequently based on MNA-SF and six times based on MNA-LF (Figure 2a,b). Participants with sarcopenia seven times more often than subjects with ‘normal’ phenotype declared problems with mobility in item C of the MNA questionnaire (able to get out of bed or chair but does not go out), and two times more frequently suffered psychological stress or acute disease in the past three months (item D; *p* = 0.026). Other differences referred to lower fluid consumption by subjects with sarcopenia in comparison with participants with a ‘normal’ phenotype (item M; *p* = 0.03), worse self-view of nutritional status (item N; *p* = 0.002), and health status in comparison with other people of the same age (item *p*; *p* = 0.009) (Table S1). Subjects with sarcopenia phenotype had the lowest scores in domains I, II, and III in the MNA-LF. Detailed characteristics are shown in Table 3.

#### 3.2.2. Obesity Phenotype

Participants with obesity had higher body weight (*p* = 0.000), BMI (*p* = 0.000), TBW (*p* = 0.003), FM (*p* = 0.000), PBF (*p* = 0.000), SMM (*p* = 0.000), FFM (*p* = 0.002) and ALM index (*p* = 0.000) in comparison with subjects with ‘normal’ phenotype (Table 2b). Upper and lower limb muscle strength in the obesity phenotype group was the highest of all groups (Table 2c), as was the nutritional status based on the MNA questionnaire; no significant differences concerning the group with ‘normal’ phenotype were observed. None of the participants with obesity had MNA-SF or MNA-LF scores indicating malnutrition, and the percentage of subjects at risk of malnutrition was two times lower than in the group with ‘normal’ phenotype, and four times lower than in sarcopenia and SO groups (Figure 2a,b). Subjects with obesity had the highest self-view of nutritional status (MNA-LF item O) and health status (item P; Table S1). They also had the highest scores in three out of four MNA-LF domains (except for domain II, in which the mean score in the obese group was equal to the ′normal′ phenotype group; Table 3).

#### 3.2.3. Sarcopenic Obesity Phenotype

Subjects with SO were the oldest of the compared groups (lack of significance compared to the sarcopenia group) and had the highest BMI, BFM, and PBF (lack of significance for subjects with obesity) (Table 2b). Compared to participants with a ′normal′ phenotype, subjects with SO had higher SMM, FFM, and ALM; however, their ALM/BMI demonstrated the highest muscle mass loss among the analyzed groups (0.5 vs. 0.7 in the sarcopenia group; *p* = 0.0018) (Table 2b). Participants in the SO group had the lowest lower limb muscle strength and physical capacity (lack of significant differences vs. sarcopenia group only) (Table 2c). Sarcopenic obesity was associated with the highest morbidity, the number of prescribed drugs, and the highest percentage of subjects with low education levels (primary or lower) (Table 2a). Subjects with SO had a four times higher prevalence of PNS than the obesity group (*p* = 0.0031) (Figure 2a,b) and the worst of all groups’ scores in MNA-LF domain IV (Table 3). They were the least likely to self-view their health status as better than other people of their age. However, one out of four subjects with SO considered it as good as other people (Table S1).

#### 3.2.4. ‘Normal’ Phenotype (Non-Sarcopenic, Non-Obese)

Only 54.7% of participants classified as ‘normal’ phenotype had good muscle mass and strength and physical capacity parameters. However, one out of five persons with a ‘normal’ phenotype had low muscle mass or strength, which was however insufficient to diagnose sarcopenia (respectively, Table 2b,c). ‘Normal’ phenotype was associated with higher education levels and fewer prescribed drugs (Table 2a). The percentage of subjects with normal nutritional status based on the MNA score was high in this group (71.7%; a higher percentage was found in the obesity group only 85.5%). However, almost one-third of subjects classified as ‘normal’ phenotype had some deviations in nutritional status (Figure 2a,b).

## 4. Discussion

Abnormal body composition is common in older adults, as demonstrated in our analysis: almost half of our study sample had sarcopenia, obesity, or SO. Moreover, one-third of participants had PNS, being another factor deteriorating health status and threatening successful aging. It should be emphasized that lack of sarcopenia or obesity, classified as a ‘normal’ (non-sarcopenic, non-obese) phenotype in our analysis, should not be considered truly normal, healthy phenotype-one out of five subjects with such phenotype in our study sample had low muscle mass or reduced muscle strength, and one out of four had malnutrition or was at risk of malnutrition. While it is true that abnormalities in muscle mass or function in these subjects were insufficient to diagnose one of the three pathological phenotypes, they may predispose to the development of pathological body composition in the future.

A literature review revealed that diagnostic methods of body composition and muscle function assessment and cut-off points used to identify sarcopenia, obesity, and SO phenotypes vary significantly between studies [48,49,50,51]. This lack of widely-accepted standards encumbers the assessment of body composition pathology prevalence and precludes elaboration of preventive and treatment strategies. Comparing our results with data from earlier studies further confirms methodological gaps in the field. For example, Khanal et al. [52] assessed older women living in the community (n = 307, mean age 71 ± 6 years). They divided participants into four groups based on body composition phenotype assessed with the BIA method, similarly to our study. However, they used different cut-off points to diagnose sarcopenia (Skeletal Muscle Index (SMI) < 6.76 kg/m^2^ for low muscle mass and HGS < 28.5 kg for low muscle strength). They used the same as us parameter to diagnose obesity (PBF), but they adopted different reference points to indicate excessive fatness (PBF > 38%). They defined SO phenotype as concomitant fulfilling the criteria mentioned above for both conditions. Subjects without sarcopenia or obesity were classified as non-sarcopenia, non-obese (healthy) phenotype. The prevalence of sarcopenia, obesity, SO, and non-sarcopenia non-obese phenotype in Khanal et al. [52] was 2.3%, 57.3%, 25.1%, and 15.3%, respectively. Obesity was twice more prevalent and SO three times more prevalent than in our study, while sarcopenia was much sparser (2.3% vs. 7.9%). These results, however, are hardly comparable, owing to different methods of muscle mass assessment (SMI vs. ALM index) and the lower cut-off points for excessive fatness (PBF 38% vs. 42%). In a recent Turkish study by Bahat et al. [53], including 1468 subjects (mean age 74.5 ± 6.9 years), the sarcopenia component in the SO phenotype was diagnosed based on the SMM/h^2^ index. The prevalence of SO phenotype was very low in this study (0.2%). However, replacing this index with SMM/BMI resulted in a 20-fold increase in the prevalence of SO (4%). We applied ALM/BMI as a sarcopenic component in diagnosing SO phenotype, following several previous studies [54,55,56]. The sarcopenia-obesity phenotype may be diagnosed less often if sarcopenia is assessed based on ALM index adjusted to height compared to ALM adjusted to body weight or BMI [57,58]. The explanation is a positive correlation between muscle mass and body weight or BMI; relatively high muscle mass in obese subjects may be inadequate for total body weight. Therefore, the lack of adjustment to body weight or BMI may lead to the underdiagnosing of sarcopenia in obese subjects [46,53]. Even if muscle mass in subjects with SO phenotype was relatively high in our study, it was the lowest among the analyzed phenotypes after adjustment to BMI.

The best method of diagnosing obesity in elderly subjects, in whom excessive fatness is not always associated with high BMI, is currently being discussed. Among numerous parameters used to diagnose obesity, such as BMI, PBF, waist circumference, waist to hip ratio (WHR), and visceral fat area, BMI is the most frequently used in clinical practice. While BMI is an important predictor of adverse health effects in adults, it has some limitations in older people. This parameter does not distinguish between FFM and FM nor assesses the amount and distribution of body fat. For these reasons, BMI may lead to a false classification of subjects with normal body mass and excessive fatness as healthy individuals [59,60,61]. Some authors demonstrated that fat tissue percentage increased in older subjects independently of body mass. Lührmann et al. [62] observed 363 women and 153 men (mean age 67.4 ± 5.9 and 66.9 ± 5.2 years, respectively) for eight years. They observed decreases in height, increases in BMI and FM, and decreases in FFM and WHR even though the body weight remained unchanged. The explanation of this phenomenon may be the masking of a decrease in FFM with an increase in fat tissue. The sensitivity of BMI ≥ 30 kg/m^2^ in the diagnosis of obesity is highest in the age range of 40–49.9 years in men (44%) and 50–59.9 years in women (54%); it decreases to 27% and 43%, respectively, in subjects aged 70–79 years [59].

Excessive body weight may also result in an underestimation of malnutrition. Being an item in the MNA questionnaire, BMI determines 10% of the total score [63]. It should be emphasized that all older persons with BMI ≥ 23 kg/m^2^ get the highest possible score for this item, which lowers the possibility of diagnosing malnutrition with the MNA questionnaire in such subjects. In line with these observations, the Global Leadership Initiative on Malnutrition (GLIM) experts assumed that BMI ≤ 22 favors malnutrition in people aged ≥70 years [64]. Other anthropometric parameters that may affect the total MNA score (to reduce the possibility of diagnosing malnutrition in obese subjects) are arm and calf circumferences (items Q and R). In our analysis, subjects with obesity and SO phenotypes had the highest scores in MNA domain I (anthropometrics), which resulted in a less frequent diagnosis of malnutrition or its risk.

Our results demonstrate the relationship between nutritional status, muscle mass, and muscle strength loss. Both subjects with sarcopenia and SO phenotype had lower MNA scores than groups with obese and ‘normal’ phenotype (the obese group received the highest score). According to the most recent GLIM recommendations, malnutrition can be diagnosed despite high BMI values if at least one other phenotypic criterion (reduced muscle mass or unintended body weight loss) and at least one etiologic criterion (reduced food intake/assimilation or disease burden/inflammatory condition) are fulfilled in a subject with a positive result of a validated screening test (e.g., MNA) [64]. This new GLIM approach presents considerable progress in the diagnostics of malnutrition concomitant with increased body mass (so-called ‘double burden’) and emphasizes the importance of muscle mass loss [65].

Reduction in muscle mass is a common component of malnutrition and sarcopenia. Malnutrition may directly contribute to sarcopenia development (resulting from inadequate delivery of calories and proteins necessary for muscle mass maintenance). Sarcopenia may, in turn, boost malnutrition (impaired mobility may increase difficulties in daily activities, such as preparing meals or buying food). Sato et al. [66] demonstrated that older persons with impaired nutritional status (malnourished or at risk of malnutrition) had a 14-times higher risk of developing sarcopenia than individuals with normal nutritional status. The results of our study confirm the association between impaired nutritional status and sarcopenia. Over 80% of participants with sarcopenia had PNS (based on the MNA-LF score, one out of five persons with sarcopenia was malnourished). In contrast, only 25% of individuals with a ′normal′ phenotype had PNS. Concomitant fulfilling the diagnostic criteria for malnutrition and sarcopenia is regarded as a malnutrition-sarcopenia syndrome, a concept proposed by Vandewounde et al. in 2012 [18].

As the definition of SO comprises two distinct phenotypes, methodological chaos in literature is not surprising. According to Batsis et al. [67], the prevalence of SO phenotype may differ 26-fold depending on the adopted definition and diagnostics criteria. Donini et al. [46] have recently recommended new diagnostic criteria for SO, which may help unify this condition’s diagnosis. According to these recommendations, diagnosis of SO should be based on reduced upper limb muscle strength (HGS) and/or lower limb muscle strength (strength of knee extensors, 5STS or Chair Stand Test) combined with abnormal body composition (low muscle mass and excessive fatness assessed with DEXA (dual-energy X-ray absorptiometry) or BIA method). In principle, our study was conducted following this approach. However, we applied the widely used ALM/BMI ratio for muscle mass assessment instead of the recently recommended ALM/Weight. We observed that the risk of malnutrition is higher in the SO phenotype compared to obesity alone. Our results align with Chang et al. [68], who demonstrated that sarcopenia has a more potent influence on nutritional status than obesity in subjects with SO phenotype. Therefore, elderly subjects with sarcopenia and obesity had poorer nutritional status than their obese counterparts. Diagnosis of SO is crucial for therapeutic interventions because considerable caloric restrictions aimed at reducing body weight in obese subjects will also lead to muscle mass loss. This effect will be particularly harmful in subjects with SO, leading to nutritional and health status deterioration.

Our study has some limitations. Body composition was assessed with the BIA method instead of computed tomography, magnetic resonance imaging, or DEXA, which are considered more precise but are hardly available in Poland. Still, EWGSOP2 recommends using bioelectric impedance in clinical practice as a reliable, cheap, and portable alternative method; it is widely used to assess the body composition of older subjects at their places. Additionally, BIA is free of X-ray exposure. We did not use the Geriatric Depression Scale (GDS) to assess the risk of depression in our study sample; instead, the participants declared neuropsychological problems in the MNA item E. Another limitation of our study was the small number of participants diagnosed with sarcopenia and SO.

Our analysis also has strong points. To the best of our knowledge, this is the first study in the field conducted in central Europe. We decided to take advantage of the MNA questionnaire, widely used in geriatrics. To this end, we analyzed the MNA total score and scores for its four domains (I—anthropometrics, II—general health status, III—dietary habits, IV—self-view of nutritional and health status). For example, domain III, assessing the intake of proteins, fluids, fruit, and vegetables, may help determine dietary habits and feeding mode, particularly important for nutritional status.

## 5. Conclusions

Abnormal body composition phenotype is prevalent in older adults. Sarcopenia and SO phenotypes are often associated with PNS. These conditions may increase the risk of adverse health consequences and augment health-related costs. Efforts should be taken to identify abnormal body composition and concomitant PNS early and elaborate therapeutic interventions. Effective prophylaxis of these conditions may improve functioning and quality of life in older people.

## Figures and Tables

**Table 1:** Characteristics of study sample by sex and age.

Variable	Total Study Sample (n = 211)	Men (n = 84)			Women (n = 127)		
		Younger Old (n = 59)	Older Old (n = 25)	*p*	Younger Old (n = 81)	Older Old (n = 46)	*p*
	Mean (SD)	Mean (SD)	Mean (SD)		Mean (SD)	Mean (SD)	
Age [years]	72.4 (7.0)	67.3 (4.1)	80.8 (3.9)	0.0000	68.9 (3.5)	80.3 (4.2)	0.0000
Height [m]	1.6 (0.1)	1.7 (0.1)	1.7 (0.1)	0.2437	1.6 (0.1)	1.6 (0.1)	0.3090
Body weight [kg]	72.7 (15.9)	82.6 (15.7)	74.8 (12.0)	0.0291	67.9 (15.4)	67.1 (12.9)	0.7563
BMI [kg/m^2^]	27.4 (5.4)	27.8 (4.9)	25.8 (4.0)	0.0716	27.5 (6.0)	27.7 (5.5)	0.8415
MNA-SF	11.9 (2.2)	12.5 (1.9)	11.9 (2.3)	0.2310	11.7 (2.6)	11.6 (1.9)	0.2511
MNA-LF	24.7 (3.6)	25.3 (2.9)	24.6 (3.8)	0.4712	24.5 (4.2)	24.5 (3.0)	0.3725
MNA Domain I	6.9 (1.8)	7.2 (1.5)	6.8 (1.9)	0.3318	6.7 (2.0)	6.8 (1.7)	0.8091
MNA Domain II	7.2 (1.4)	7.5 (1.3)	7.1 (1.3)	0.2706	7.3 (1.5)	7.0 (1.4)	0.1421
MNA Domain III	7.8 (1.1)	7.8 (0.9)	7.6 (1.0)	0.3454	7.8 (1.2)	7.9 (1.0)	0.9549
MNA Domain IV	2.8 (1.0)	2.8 (0.9)	3.0 (1.0)	0.3038	2.7 (1.1)	2.8 (1.1)	0.5479
	n (%)	n (%)	n (%)		n (%)	n (%)	
Marital status							
Unmarried	95 (46.1)	11 (19.0)	10 (40.0)	0.0431	43 (55.8)	31 (67.4)	0.2056
Married	111 (53.9)	47 (81.0)	15 (60.0)		34 (44.2)	15 (32.6)	
Residential pattern ^a^							
Living alone	70 (34.0)	7 (12.1)	6 (24.0)	0.2970	31 (40.3)	26 (56.5)	0.0801
Living with a mate	136 (66.0)	51 (87.9)	19 (76.0)		46 (59.7)	20 (43.5)	
Education level ^b^							
Lower than primary or primary	14 (6.8)	3 (5.2)	1 (4.0)	0.7416	4 (5.2)	6 (13.3)	0.2153
Higher than primary	191 (93.2)	55 (94.8)	24 (96.0)		73 (94.8)	39 (86.7)	
MNA-SF							
Malnutrition	13 (6.2)	2 (3.4)	2 (8.0)	0.4871	7 (8.6)	2 (4.3)	0.1105
At risk of malnutrition	49 (23.2)	8 (13.6)	5 (20.0)		18 (22.2)	18 (39.1)	
Normal nutritional status	149 (70.6)	49 (83.1)	18 (72.0)		56 (69.1)	26 (56.5)	
MNA-LF							
Malnutrition	7 (3.3)	0 (0.0)	1 (4.0)	0.2882	6 (7.4)	0 (0.00	0.0133
At risk of malnutrition	59 (28.0)	16 (27.1)	7 (28.0)		18 (22.2)	18 (39.1)	
Normal nutritional status	145 (68.7)	43 (72.9)	17 (68.0)		57 (70.4)	28 (60.9)	
Nutritional status							
PNS	66 (31.3)	16 (27.1)	8 (32.0)	0.6507	24 (29.6)	18 (39.1)	0.2740
Normal nutritional status	145 (68.7)	43 (72.9)	17 (68.0)		57 (70.4)	28 (60.9)	

Notes: ^a^ data missing for 5 subjects; ^b^ data missing for 6 subjects. Abbreviations: n, number; SD; Standard Deviation; BMI, Body Mass Index; MNA-SF, Mini Nutritional Assessment-Short Form; MNA-LF, Mini Nutritional Assessment; PNS, Poor Nutritional Status; *p*, significance level.

**Table 2:** (a). Socio-demographic parameters, health, nutritional and functional status by phenotype groups. (b). Anthropometric characteristics and body composition parameters by phenotype groups. (c). Physical fitness parameters by phenotype groups.

**(a)**					
**Variable**	**Sarcopenia** **(n = 21)**	**Obesity** **(n = 69)**	**SO** **(n = 15)**	**Non-Sarcopenic** **Non-Obese** **(n = 106)**	***p***
Sex					
Women	10 (47.62)	34 (49.28)	10 (66.67)	73 (68.87)	0.1351
Men	11 (52.38)	35 (50.72)	5 (33.33)	33 (31.13)	
Age cohorts					
Younger old	11 (52.38)	52 (75.36)	5 (33.33)	72 (67.92)	0.0378
Older old	10 (47.62)	17 (24.64)	10 (66.67)	34 (32.08)	
Marital status ^a^					
Unmarried	9 (47.4)	25 (36.8)	10 (66.7)	51 (49.0)	0.1503
Married	10 (52.6)	43 (63.2)	5 (33.3)	53 (51.0)	
Residential pattern ^a^					
Living alone	6 (31.6)	20 (29.4)	5 (33.3)	39 (37.5)	0.7390
Living with a mate	13 (68.4)	48 (70.6)	10 (66.7)	65 (62.5)	
Education level ^b^					
Lower than primary or primary	1 (5.6)	5 (7.4)	5 (33.3)	3 (2.9)	0.0060
Higher than primary	17 (94.4)	63 (92.6)	10 (66.7)	101 (97.1)	
Number of chronic diseases ^a^ [mean (SD)]	3.7 (1.6)	3.1 (1.6)	5.1 (2.2)	3.1 (1.7)	0.0043
Number of prescribed drugs ^c^ [mean (SD)]	6.2 (3.3)	6.0 (3.7)	9.5 (4.0)	5.2 (4.0)	0.0013
AMTS [mean (SD)]	9.1 (0.7)	9.5 (0.6)	9.2 (0.9)	9.4 (0.6)	0.0736
ADL ^d^ [mean (SD)]	5.7 (0.3)	5.9 (0.3)	5.2 (0.8)	5.8 (0.3)	0.0000
IADL ^d^ [mean (SD)]	23.5 (2.5)	26.0 (1.7)	21.1 (4.0)	25.8 (2.1)	0.0000
MNA-SF [mean (SD)]	9.2 (3.0)	12.8 (1.3)	11.6 (1.9)	11.9 (2.2)	0.0000
MNA-LF[mean (SD)]	20.2 (4.5)	26.1 (2.1)	23.4 (2.7)	24.9 (3.5)	0.0000
**(b)**					
**Variable**	**Sarcopenia** **(n = 21)**	**Obesity** **(n = 69)**	**SO** **(n = 15)**	**Non-Sarcopenic** **Non-Obese** **(n = 106)**	***p***
Age [years]	74.5 (8.2)	70.3 (6.4)	78.9 (6.0)	72.3 (6.6)	0.0002
Height [m]	1.6 (0.1)	1.6 (0.1)	1.6 (0.1)	1.6 (0.1)	0.0053
Body weight [kg]	54.9 (11.7)	85.8 (11.8)	82.3 (11.0)	66.3 (12.1)	0.0000
BMI [kg/m^2^]	20.9 (3.0)	31.6 (3.4)	34.2 (5.1)	25.0 (3.6)	0.0000
Mid-arm circumference [cm] ^e^	22.35 (2.71)	30.08 (2.92)	32.11 (5.28)	26.33 (3.29)	0.0000
Calf circumference [cm] ^f^	30.56 (3.80)	38.84 (3.23)	38.35 (3.03)	35.03 (3.53)	0.0000
TBW [l]	29.7 (5.7)	37.7 (6.9)	33.4 (5.1)	34.0 (6.9)	0.0000
FM [kg]	14.6 (5.8)	34.6 (7.1)	37.0 (6.9)	20.4 (6.7)	0.0000
PBF [%]	25.2 (7.2)	40.4 (6.6)	44.9 (4.8)	30.3 (7.8)	0.0000
SMM [kg]	21.5 (4.5)	28.2 (5.5)	24.6 (4.1)	25.2 (5.6)	0.0000
FFM [kg]	40.3 (7.6)	51.2 (9.3)	45.2 (6.9)	45.9 (9.8)	0.0000
ALM upper limbs [kg]	3.9 (1.2)	5.9 (1.5)	5.2 (1.2)	4.8 (1.6)	0.0000
ALM lower limbs [kg]	11.6 (3.0)	15.2 (3.2)	12.9 (2.3)	13.5 (3.2)	0.0000
ALM [kg]	15.4 (4.1)	21.1 (4.6)	18.1 (3.4)	18.4 (4.7)	0.0000
ALM index [kg/m^2^]	5.8 (1.0)	7.7 (1.0)	7.5 (1.0)	6.8 (1.10	0.0000
ALM/BMI	0.7 (0.2)	0.7 (0.2)	0.5 (0.1)	0.7 (0.2)	0.0001
Low muscle mass as low ALM index [n (%)]	21 (100.0)	5 (7.2)	2 (13.3)	21 (19.8)	0.0000
Low muscle mass as low ALM/BMI [n (%)]	3 (14.3)	23 (33.3)	15 (100.0)	9 (8.5)	0.0000
(c)					
Variable	Sarcopenia (n = 21)	Obesity (n = 69)	SO (n = 15)	Non-Sarcopenic Non-Obese (n = 106)	*p*
The best of four upper limb strength measurements [kg]	18.9 (4.20)	31.7 (9.8)	17.0 (6.0)	27.2 (9.5)	0.0000
Five-Repetition Sit-to-Stand test [s] ^g^	18.0 (7.3)	12.1 (4.5)	17.6 (4.9)	12.4 (4.3)	0.0000
4 m usual walking speed test [m/s]	0.7 (0.3)	1.1 (0.3)	0.6 (0.3)	1.0 (0.4)	0.0000
Reduced upper limb muscle strength [n (%)]	16 (76.2)	0 (0.0)	12 (80.0)	7 (6.6)	0.0000
Reduced lower limb muscle strength ^g^ [n (%)]	12 (63.2)	8 (11.6)	8 (66.7)	20 (19.4)	0.0000
Reduced muscle strength according EWGSOP2 (upper and/or lower limbs) [n (%)]	21 (100.0)	8 (11.6)	15 (100.0)	22 (20.8)	0.0000
Low gait speed [n (%)]	12 (57.1)	10 (14.5)	11 (73.3)	19 (17.9)	0.0000

Notes: (a) Data are presented as n (%) unless otherwise indicated. ^a^ data missing for 5 subjects; ^b^ data missing for 6 subjects; ^c^ data missing for 8 subjects; ^d^ data missing for 2 subjects. Abbreviations: n, number; SO, Sarcopenic Obesity; SD, Standard deviation, AMTS, Abbreviated Mental Test Score; ADL, Activities of Daily Living; IADL, Instrumental Activities of Daily Living; MNA-SF, Mini Nutritional Assessment-Short Form; MNA-LF, Mini Nutritional Assessment-Long Form; *p*, significance level. (b) Data are presented as mean (SD) unless otherwise indicated. ^e^ data missing for 35 subjects; ^f^ data missing for 32 subjects. Abbreviations: n, number; SO, Sarcopenic Obesity; SD, Standard deviation, BMI, Body Mass Index; TBW, Total Body Water; FM, Fat Mass; PBF, Percent Body Fat; SMM, Skeletal Muscle Mass; FFM, Fat-Free Mass; ALM, Appendicular Lean Mass; *p*, significance level. (c) Data are presented as mean (SD) unless otherwise indicated. ^g^ data missing for 8 subjects. Abbreviations: n, number; EWGSOP2, extended group for the European Working Group on Sarcopenia in Older People; *p*, significance level.

**Table 3:** Mini Nutritional Assessment (MNA) domains by phenotype groups.

MNA Domains	Total (n = 211)	Sarcopenia (n = 21)	Obesity (n = 69)	Sarcopenic Obesity (n = 15)	Non-Sarcopenic Non-Obese (n = 106)	
	Mean (SD)	Mean (SD)	Mean (SD)	Mean (SD)	Mean (SD)	*p*
Domain I—anthropometrics						
B. Weight loss during the last 3 months	2.5 (0.9)	2.1 (1.1)	2.6 (0.8)	2.5 (1.0)	2.4 (0.9)	0.1400
F. Body Mass Index	2.6 (0.8)	1.5 (1.1)	3.0 (0.0)	3.0 (0.0)	2.6 (0.9)	0.0000
Q. Mid-arm circumference in cm	0.9 (0.3)	0.6 (0.4)	1.0 (0.1)	1.0 (0.0)	0.9 (0.3)	0.0000
R. Calf circumference in cm	0.9 (0.3)	0.4 (0.5)	1.0 (0.0)	1.0 (0.0)	0.9 (0.3)	0.0000
Domain I (score)	6.9 (1.8)	4.6 (2.5)	7.6 (0.8)	7.5 (1.0)	6.8 (1.8)	0.0000
Domain II—general health status						
C. Mobility	1.9 (0.3)	1.7 (0.5)	2.0 (0.2)	1.7 (0.5)	2.0 (0.2)	0.0000
D. Has suffered psychological stress or acute disease in the past 3 months?	1.2 (1.0)	0.5 (0.9)	1.3 (1.0)	1.1 (1.0)	1.3 (1.0)	0.0039
E. Neuropsychological problems	1.9 (0.5)	1.9 (0.5)	1.9 (0.3)	1.4 (0.9)	1.9 (0.4)	0.0017
G. Lives independently (not in nursing home or hospital)	1.0 (0.0)	1.0 (0.0)	1.0 (0.0)	1.0 (0.0)	1.0 (0.0)	1.0000
H. Takes more than 3 prescription drugs per day	0.3 (0.5)	0.1 (0.3)	0.3 (0.5)	0.0	0.4 (0.5)	0.0009
I. Pressure sores or skin ulcers	0.9 (0.2)	0.9 (0.3)	0.9 (0.2)	0.9 (0.4)	1.0 (0.2)	0.5452
Domain II (score)	7.3 (1.4)	6.1 (1.4)	7.5 (1.2)	6.1 (1.4)	7.5 (1.3)	0.0000
Domain III—dietary habits						
A. Has food intake declined over the past 3 months due to loss of appetite, digestive problems, chewing or swallowing difficulties?	1.8 (0.5)	1.6 (0.7)	1.9 (0.3)	1.9 (0.3)	1.8 (0.5)	0.0254
J. How many full meals does the patient eat daily	1.9 (0.3)	1.9 (0.3)	1.9 (0.4)	1.9 (0.4)	1.9 (0.3)	0.9357
K. Selected consumption markers for protein intake	0.5 (0.4)	0.6 (0.4)	0.5 (0.4)	0.5 (0.4)	0.5 (0.4)	0.8823
L. Consumes two or more servings of fruit or vegetables per day?	0.8 (0.4)	0.6 (0.5)	0.8 (0.4)	0.9 (0.4)	0.8 (0.4)	0.3300
M. How much fluid (water, juice, coffee, tea, milk...) is consumed per day?	0.8 (0.3)	0.7 (0.3)	0.8 (0.2)	0.7 (0.3)	0.8 (0.3)	0.0205
N. Mode of feeding	2.0 (0.1)	2.0 (0.2)	2.0 (0.0)	2.0 (0.0)	2.0 (0.1)	0.2574
Domain III (score)	7.7 (1.1)	7.3 (1.4)	7.9 (0.8)	7.8 (0.9)	7.8 (1.1)	0.3518
Domain IV—self-view of nutritional and health status						
O. Self-view of nutritional status	1.7 (0.6)	1.6 (0.8)	1.9 (0.4)	1.4 (0.7)	1.7 (0.6)	0.3240
P. In comparison with other people of the same age, how does the patient consider his/her health status?	1.1 (0.8)	0.7 (0.9)	1.2 (0.7)	0.7 (0.7)	1.1 (0.8)	0.0081
Domain IV (score)	2.8 (1.0)	2.3 (1.2)	3.1 (0.9)	2.1 (1.1)	2.8 (1.0)	0.0011

## Data Availability

All relevant data are within the manuscript and are openly available in the Zenodo repository (DOI: 10.5281/zenodo.6624902).

## Supplementary Materials

The following supporting information can be downloaded at: https://www.mdpi.com/article/10.3390/nu14142889/s1, Table S1: Mini Nutritional Assessment (MNA) questionnaire items by phenotype groups.
