# Supplementary material for: Sarcopenia, Obesity, Sarcopenic Obesity and Risk of Poor Nutritional Status in Polish Community-Dwelling Older People Aged 60 Years and Over
Source: Nutrients. 2022 Jul 14;14(14):2889. doi: 10.3390/nu14142889 (PMC9317847; doi:10.3390/nu14142889)
Supplement: Supplementary file 1 [file nutrients-14-02889-s001.zip › nutrients-1787440-supplementary.pdf]

Table S1. Mini Nutritional Assessment (MNA) questionnaire items by phenotype groups.

MNA items	Total (n=211)	Sarcopenia (n=21)	Obesity (n=69)	Sarcopenic Obesity (n=15)	Non-sarcopenic non-obese (n=106)	
	n (%)	n (%)	n (%)	n (%)	n (%)	p
**Domain I - anthropometrics**						
**B. Weight loss during the last 3 months**						
No weight loss	142 (67.3)	11 (52.4)	52 (75.4)	11 (73.3)	68 (64.2)	0.4093
Weight loss between 1 and 3 kg	40 (19.0)	4 (19.0)	11 (15.9)	1 (6.7)	24 (22.6)	
Does not know	14 (6.6)	3 (14.3)	3 (4.3)	2 (13.3)	6 (5.7)	
Weight loss greater than 3 kg	15 (7.1)	3 (14.3)	3 (4.3)	1 (6.7)	8 (7.5)	
**F. Body Mass Index (BMI)**						
BMI <19	10 (4.7)	4 (19.0)	0	0	6 (5.7)	0.0000
19 ≤BMI < 21	17 (8.1)	8 (38.1)	0	0	9 (8.5)	
21≤BMI <23	13 (6.2)	3 (14.3)	0	0	10 (9.4)	
BMI ≥23	171 (81.0)	6 (28.6)	69 (100.0)	15 (100.0)	81 (76.4)	
**Q. Mid-arm circumference (MAC) in cm**						
MAC <21	12 (5.7)	6 (28.6)	0	0	6 (5.7)	0.0000
MAC 21 to 22	10 (4.7)	4 (19.0)	1 (1.4)	0	5 (4.7)	
MAC > 22	189 (89.6)	11 (52.4)	68 (98.6)	15 (100.0)	95 (89.6)	
**R. Calf circumference (CC) in cm**						
CC < 31	23 (10.9)	13 (61.9)	0	0	10 (9.4)	0.0000
CC ≥ 31	188 (89.1)	8 (38.1)	69 (100.0)	15 (100.0)	96 (90.6)	
**Domain II – general health status**						
**C. Mobility**						
Goes out	195 (92.4)	15 (71.4)	67 (97.1)	11 (73.3)	102 (96.2)	0.0003
Able to get out of bed/chair but does not go out	16 (7.6)	6 (28.6)	2 (2.9)	4 (26.7)	4 (3.8)	
Bed or chair bound	0	0	0	0	0	
**D. Has suffered psychological stress or acute disease in the past 3 months?**						
No	126 (59.7)	5 (23.80	46 (66.7)	8 (53.3)	67 (63.2)	0.0038
Yes	85 (40.3)	16 (76.2)	23 (33.30	7 (46.7)	39 (36.8)	
**E. Neuropsychological problems**						
No psychological problems	194 (91.9)	19 (90.5)	66 (95.7)	10 (66.7)	99 (93.4)	0.0729
Mild dementia	7 (3.3)	1 (4.8)	2 (2.9)	1 (6.7)	3 (2.8)	
Severe dementia or depression	10 (4.7)	1 (4.8)	1 (1.4)	4 (26.7)	4 (3.8)	
**G. Lives independently (not in nursing home or hospital)**						
Yes	211 (100.0)	21 (100.0)	69 (100.0)	15 (100.0)	106 (100.0)	
No	0	0	0	0	0	
**H. Takes more than 3 prescription drugs per day**						
Yes	144 (68.2)	19 (90.5)	48 (69.6)	15 (100.0)	62 (58.5)	0.0001
No	67 (31.8)	2 (9.5)	21 (30.4)	0	44 (41.5)	
**I. Pressure sores or skin ulcers**						
Yes	13 (6.2)	2 (9.5)	4 (5.8)	2 (13.3)	5 (4.7)	0.6135
No	198 (93.8)	19 (90.5)	65 (94.2)	13 (86.7)	101 (95.3)	
**Domain III – dietary habits**						
**A. Has food intake declined over the past 3 months due to loss of appetite, digestive problems, chewing or swallowing difficulties?**						
No decrease in food intake	178 (84.4)	14 (66.7)	63 (91.3)	14 (93.3)	87 (82.1)	0.0662

Moderate decrease in food intake	27 (12.8)	5 (23.8)	6 (8.7)	1 (6.7)	15 (14.2)	
Severe decrease in food intake	6 (2.8)	2 (9.5)	0	0	4 (3.8)	
**J. How many full meals does the patient eat daily?**						
1 meal	0	0	0	0	0	0.9361
2 meals	24 (11.4)	2 (9.5)	9 (13.0)	2 (13.3)	11 (10.4)	
3 meals	187 (88.6)	19 (90.5)	60 (87.0)	13 (86.7)	95 (89.6)	
**K. Selected consumption markers for protein intake**						
**– at least serving of dairy products per day (data missing from 13 subjects)**						
Yes	152 (76.8)	16 (76.2)	48 (75.0)	9 (64.3)	79 (79.8)	0.6266
No	46 (23.2)	5 (23.8)	16 (25.0)	5 (35.7)	20 (20.2)	
**– two or more servings of legumes or eggs per week (data missing from 13 subjects)**						
Yes	163 (82.3)	17 (81.0)	53 (82.8)	9 (64.3)	84 (84.8)	0.3810
No	35 (17.7)	4 (19.0)	11 (17.2)	5 (35.7)	15 (15.2)	
**- meat, fish or poultry every day (data missing from 13 subjects)**						
Yes	98 (49.5)	12 (57.1)	32 (50.0)	7 (50.0)	47 (47.5)	0.8824
No	100 (50.5)	9 (42.9)	32 (50.0)	7 (50.0)	52 (52.5)	
**L. Consumes two or more servings of fruit or vegetables per day?**						
No	50 (23.7)	8 (38.1)	15 (21.7)	2 (13.3)	25 (23.6)	0.3449
Yes	161 (76.3)	13 (61.9)	54 (78.3)	13 (86.7)	81 (76.4)	
**M. How much fluid (water, juice, coffee, tea, milk...) is consumed per day?**						
< 3 cups	5 (2.4)	1 (4.8)	0	1 (6.7)	3 (2.8)	0.0364
3 to 5 cups	77 (36.5)	12 (57.1)	26 (37.7)	8 (53.3)	31 (29.2)	
> 5 cups	129 (61.1)	8 (38.1)	43 (62.3)	6 (40.0)	72 (67.9)	
**N. Mode of feeding**						
Unable to eat without assistance	0	0	0	0	0	0.3536
Self-fed with some difficulty	2 (0.9)	1 (4.8)	0	0	1 (0.9)	
Self-fed without any problem	209 (99.1)	20 (95.2)	69 (100.0)	15 (100.0)	105 (99.1)	
**Domain IV self-view of nutritional and health status**						
**O. Self view of nutritional status**						
Views as being malnourished	11 (5.2)	4 (19.0)	0	2 (13.3)	5 (4.7)	0.0020
Is uncertain of nutritional state	39 (18.5)	1 (4.8)	10 (14.5)	5 (33.3)	23 (21.7)	
Views self as having no nutritional problem	161 (76.3)	16 (76.2)	59 (85.5)	8 (53.3)	78 (73.6)	
**P. In comparison with other people of the same age, how does the patient consider his/her health status?**						
Not as good	40 (19.0)	10 (47.6)	7 (10.1)	4 (26.7)	19 (17.9)	0.0089
Does not know	41 (19.4)	4 (19.0)	15 (21.7)	5 (33.3)	17 (16.0)	
As good	51 (24.2)	1 (4.8)	17 (24.6)	4 (26.7)	29 (27.4)	
Better	79 (37.4)	6 (28.6)	30 (43.5)	2 (13.3)	41 (38.7)	
